# The range and diversity of providers’ viewpoints towards the Iraqi primary health care system: an exploration using Q-methodology

**DOI:** 10.1186/1472-698X-13-18

**Published:** 2013-03-21

**Authors:** Nazar P Shabila, Namir G Al-Tawil, Tariq S Al-Hadithi, Egbert Sondorp

**Affiliations:** 1 Department of Community Medicine, College of Medicine, Hawler Medical University, Erbil, Iraq; 2 London School of Hygiene and Tropical Medicine, London, UK

**Keywords:** Primary health care, Q-methodology, Providers, Assessment

## Abstract

**Background:**

The increasingly recognized need for reorganizing the primary health care services in Iraq calls for a comprehensive assessment of the system to better understand its problems and needs for development. As part of such comprehensive assessment and due to the important role of primary health care providers in adopting any change, we ought to explore the range and diversity of viewpoints of primary health care providers towards the Iraqi primary health care system.

**Methods:**

This explorative study was carried out in Erbil governorate, Iraq from May to July 2011. Data were collected from primary health care providers using Q-methodology to elicit subjective viewpoints and identify shared patterns among individuals. Forty primary health care providers representing eight primary health care centers sorted 41 statements reflecting different aspects of the Iraqi primary health care system into a distribution on a scale of nine from “disagree most” to “agree most”. By-person factor analysis was used to derive latent viewpoints through centroid factor extraction and varimax rotation of factors.

**Results:**

Analysis of the participants’ Q-sorts resulted in four distinct viewpoints among primary health care providers toward the current primary health care system. One factor emphasized positive aspects of the current primary health care system that is content with the current primary health care system. The other three factors highlighted the negative aspects and they included (i) professionally-centered viewpoint, (ii) comprehensive perception and problem-based solutions and (iii) critical to leadership/governance aspects of the system.

**Conclusions:**

This study revealed diverse viewpoints of primary health care providers toward the current Iraqi primary health care system and recognized the particular issues related to each viewpoint. The findings can contribute to a better understanding of health policy makers and primary health care managers concerning the problems facing the primary health care system that might contribute to change in the management of this system.

## Background

Conflicts, social unrest and political instability can significantly affect the ability of a health care system to meet the needs of its population. Disruption of health services and even collapse of the health system, in particular primary health care (PHC) component, from the effect of conflicts and political instability have been well-documented in different settings [1,2].

The prolonged conflict from the different wars, international sanctions, the ensuing major disruptions, factional fighting and political instability during the last few decades have deeply affected Iraq’s society, including its health sector [3]. These events left a crippled health system struggling to meet population needs [4,5]. Years after the 2003 war, the Iraqi health system still lies in a fragile status. Thousands of doctors have fled the country, the major health indicators witnessed a substantial fall, the unmet mental health needs have increased and the pace of heath care reconstruction and reform remains slow [6].

The PHC system did not escape these damaging effects and continues to suffer from problems common throughout the health care system [3,7]. About 40% of the country’s PHC centers lack physicians [6]. The functional components of the PHC system require heavy investment to meet the population needs. The system suffers from inappropriate service delivery with shortage in supplies and necessary equipment leading to increased and unnecessary referrals to hospitals. There are poorly organized patient records with limited use of information technology. No mechanisms exist to ensure a minimum level of quality of care [8,9]. Accreditation and licensing systems are outdated and un-enforced, with no requirements for continuing medical education [8,10].

The PHC services in Iraq are provided by a network of public PHC centers that are of two types. The main PHC centers that are located in main urban and semi-urban areas are staffed by one or more physicians in addition to a number of nurses, medical assistants and admin/support staff. The smaller PHC centers or dispensaries are located in rural areas and are staffed by non-physician providers, usually nurses or medical assistants [10]. The PHC providers receive regular fixed salaries from the state. The salary is not related to the number of patients visiting the center or the consultations fees [5]. People have open access to PHC centers with the payment of 250 Iraqi Dinars (0.20 US$) and receive free drugs under severe restrictions in quantities [10]. They can drop in any PHC center of their preference without having previous registration or made appointment. The physicians at the PHC centers are mainly general practitioners. General practitioners in Iraq are physicians who lack higher qualification after graduation from medical school, apart from a two years clinical internship and serving one year in rural areas. With the increasing number of specialists in Iraq during the last few years and the limited availability of working places for them in hospitals and consultancy centers, more and more specialists are assigned to work in PHC centers particularly those located in city center [5,7]. The specialty of primary care or family medicine was almost non-existent until recent introduction of family medicine specialty. Recently increased emphasis has been placed over dedicating PHC centers to family medicine taking into consideration the positive experience of a number of countries like Bahrain and Turkey on establishing this system and the benefits observed from a number of pilot projects in Iraq. There is a general belief in Iraq that establishment of family medicine model in PHC centers will result in improved health care for the general population through organizing PHC services by identification of catchment areas, registration of people with specific family units and collecting epidemiologic information [11,12].

The private health sector in Iraq provides mainly curative health services and consists of a number of relatively small surgical hospitals, many physicians’ clinics and private pharmacies. The private sector is steadily growing over the last decade. Well spread over the country, there are also many nurses and medicals assistants’ private clinics that prescribe and sell most kinds of medicines. In fact, regulations that prohibit selling drugs without prescription are poorly implemented. There is no clear separation between the public and private health sectors in Iraq as most of the health providers work in the public sector in the morning and in the private sector in the evening leading frequently to conflicts of interest [7,13].

There is a general consensus among professionals and policy makers on the poor functioning of the Iraqi PHC system and the desperate need for reorganizing and restructuring the PHC services [4,7]. However, there is limited research evidence about the specific challenges facing the system. Therefore, comprehensive assessment of the PHC system in Iraq is essential to better understand the problems facing the system and the needs for its improvement. As part of such comprehensive assessment, obtaining PHC providers’ viewpoints on the different aspects of the PHC system is critical and constitutes a corner stone for any improvement in the system due to the important role and power they have in running and adapting this system [14,15].

This study is a component of a comprehensive assessment of the PHC system in Erbil governorate, Iraq. The overall aim of the assessment of the PHC system was to systematically explore the problems the system faces and the opportunities and barriers for improvement from the perspectives of different actors in the PHC system. This component was carried out specifically to explore the range and diversity of viewpoints of PHC providers towards the Iraqi PHC system.

## Methods

### Setting

This study was carried out on a sample of PHC providers selected from eight main PHC centers located in urban and semi-urban areas of Erbil governorate of Iraq from May to July 2011. These PHC centers were randomly selected from a sample frame of all main PHC centers within Erbil governorate, stratified by their geographical locations. Three PHC centers were selected from Erbil city center (urban area), another three PHC centers from district and sub-district centers close to Erbil city (semi-urban areas within 60 Km from Erbil city), and two PHC centers from district and sub-district centers remote from Erbil city (semi-urban areas more than 60 Km away from Erbil city). Providers working in the smaller PHC centers or dispensaries located in rural areas were not included in this study.

### Q-methodology

Q-methodology, or by-person factor analysis, provides a foundation for the systematic study of subjectivity [16,17]. Uniquely, Q-methodology is a quantitative research technique that measures and quantifies traditionally qualitative data such as perceptions and attitudes [17,18]. Typically, in a Q methodological study people are presented with a sample of statements about some topic, called the Q-set. The Q-set is usually developed from different sources such as interviewing people, participant observations, expert opinions, scientific literature review and popular literature like media reports, newspapers and magazines [16,19]. Respondents, called the P-set, usually are asked to rank-order the statements from their individual point of view, according to some preference, judgment or feeling about them, mostly using a quasi-normal distribution. By Q sorting, people give their subjective meaning to the statements, and by doing so reveal their subjective viewpoint. Then individual rankings (or viewpoints) are subjected to factor analysis [16,20].

### Participants

Participants in this study were not randomly selected. In studies using Q-methodology, samples are carefully selected rather than randomized so that variability in a specific case or situation can be analyzed [21]. The selection process involved purposive sampling with the aim to represent people with different professions, having long PHC experience and from different units of the PHC center. Individuals who were potentially representative of various issues of the PHC system and those who could provide the best insights on the relevant topic were recruited. At each PHC center, five PHC providers were selected and were invited to participate in the study; a physician, a dentist or pharmacist if available, two or three trained health care workers and an administrator. The study was not subjected to sample size estimation because Q-methodology is a kind of exploratory factor analysis, not designed for hypothesis testing. The number of participants is usually, but not necessarily, smaller than the Q set [22]. The aim is usually to have four or five persons defining each anticipated viewpoint, which are often two to four, and rarely more than six. Accordingly, breadth and diversity of viewpoints is probably best achieved when a participant group contains between 40 and 60 participants [23]. Therefore a sample size of 40 persons was selected.

### Identification of statements

A pool of statements that could potentially describe and sufficiently represent the topic of investigation was generated. An open-ended questionnaire survey for assessing the Iraqi PHC system was conducted that involved 46 PHC managers, policy makers and academics [24]. Statements made by the participants about their feelings and experience with the positive aspects, problems, priority needs and barriers to improve the PHC system were obtained. Four focus groups involving 40 PHC physicians, nurses and administrative directors form 12 PHC centers were also conducted. Statements made by the participants about problems facing the PHC system and priority needs for its development were obtained to be used in this Q-study [8]. Additional statements were obtained from reviewing relevant literature about the PHC system in Iraq [25] and Serbia which had a similar post-conflict setting [2].

### Compiling the Q-sample statements

As a result of the statement identification step, 230 statements were extracted representing statements of opinion, relevant to the research questions, made by 86 respondents. A modified version of the WHO conceptual framework of health system building blocks [26] was used to group the statements under different themes of service delivery, health workforce, information, medical products, financing and leadership and governance. All the statements were reviewed for similarities and differences. Responses that were repeated were discarded, some statements of close similarity were merged and views which were polar opposite were deleted. Two members of the research team made independent decisions about these responses. The aim was to include statements from various aspects of the different themes of the WHO conceptual framework. The two researchers compared their results and discussed responses which lacked agreement until consensus was reached. The actual expressions of the respondents were used; only the grammar of several statements was edited. Finally, 41 statements were selected.

### Creating the Q-sort

**Figure 1:**
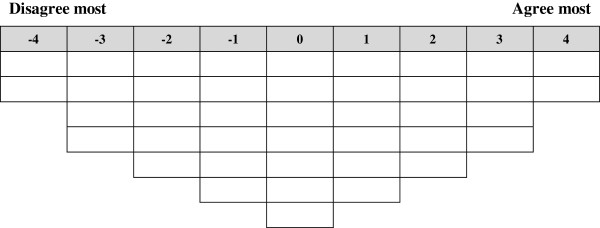
Empty score sheet used by respondents to sort the 41 statements.

The selected 41 statements were originally made in Kurdish language. Then they were translated into English language; the translation was validated by an independent Kurdish native fluent in English language, who translated the Kurdish version back to English to ensure accuracy. The statements were numbered randomly and typed onto small cards with one statement per card. The Q-sample was developed in both Kurdish and English languages so that participants could make a choice and use the language they felt most comfortable with. After the Q-sample was created, a Q-sort grid was developed, which involved creating a quasi-normal distribution with 41 cells equal to the number of the Q-sample statements (Figure 1). This Q-sort constituted the data collection instrument for the study. Both language versions of the Q-sort were pilot-tested with a convenience sample of seven PHC providers. Feedback was collected on the clarity of statements, ease of the task, length of time for completion and general suggestions about the process. As a result, modifications were made to the instrument and the instructions.

### Data collection

The purpose of the study and instructions for completing the task were explained to each selected participant and informed consent was obtained. Participants were asked to sort the cards into nine piles, from -4 (disagree most) to +4 (agree most), in relation to their perception about different aspects of the PHC system and according to the Q-sort table. Clear step by step instructions were provided to the participants about how to sort the cards and they were left to complete the sorting alone. Participants were free to choose the language of response where 13 participants (all physicians) chose to respond in English language. The study was approved by the Research Ethics Committee of Hawler Medical University and London School of Hygiene and Tropical Medicine.

### Data analysis

The PQMethod 2.11 program was used for the analysis of Q-sorts [27]. The prominent common viewpoints, known as factors, were extracted using centroid factor extraction and varimax rotation. Factors representing at least two defining sorts and having eigenvalues greater than one were extracted. Defining sorts are Q sorts which are both significant for the factor but not significant on any other factor. A conservative significance level of p < 0.01 was chosen for factor loading. Thus, those Q sorts that achieved a factor loading of 0.403 or above on a given factor were considered to have loaded significantly onto that factor [9] - an explanation of how this is calculated is shown Additional file 1. An eigenvalue is the sum of squared loadings for a factor; it conceptually represents the amount of variance accounted for by a factor [28]. However, several different factor solutions were examined for obtaining the most meaningful, consistent and coherent factors. Rotation began with seven factors - those with eigenvalues of above 1.0. However, examination of solutions provided with rotation of seven, six and five factors proved unsatisfactory, as the factors produced lacked clarity or definable attributes, resulted in large number of sorts that have not loaded significantly on any factor or those loaded significantly on two or more factors (confounding), or resulted in only single participant loading significantly on certain factor. Extraction of four factors provided the clearest solution, accounting for 44% of the variance in the correlation matrix.

Before describing and interpreting the factors, the statements factor scores and difference scores were calculated. A statement’s factor score is the normalized weighted average statement score (Z-score) of respondents that define that factor. Based on their Z-scores, statements were attributed to the original quasi-normal distribution, resulting in a composite (or idealized) Q sort for each factor. The composite Q sort of a factor represents how a hypothetical respondent with a 100% loading on that factor would have ordered all the statements of the Q-set. The statements of a factor that are located at both extremes in the composite sort are called the characterizing statements [16]. The characterizing statements in this study included those with a rank value of ^′^ + 4^′^, ^′^ + 3^′^, ^′^-3^′^, ^′^-4^′^. The difference score is the magnitude of difference between a statement’s score on any two factors that is required for it to be statistically significant. When a statement’s score on two factors exceeds this difference score, it is called a distinguishing statement. A distinguishing statement for a factor is a statement whose score on that factor is statistically significantly different from its score on any other factor [16]. Distinguishing statements that are significant at p < 0.05 are highlighted with asterisk (*), and those at p < 0.01 are highlighted with double asterisk (**) in the Results section of this study. A statement that is not distinguishing between any of the identified factors is called a consensus statement.

The resultant factors represent sorts made by individuals who have responded in essentially the same way. Each factor or viewpoint was interpreted subjectively by examining the characterizing and distinguishing statements. Finally a conceptual interpretation was developed to capture the essence of the viewpoints being endorsed. The modified version of the WHO conceptual framework of health system building blocks [26] was used wherever applicable to assist in factor interpretation.

## Results

Forty PHC providers participated in the study. Their mean ± SD age was 35.4 ± 8.9 years with a median experience with the PHC system of 6 years (range 1 to 25 years). Details of the participants’ gender and professional characteristics are shown in Table 1.

**Table 1:** Gender and professional characteristics of the participants

**Characteristics**	**No.**	**(%)**
**Gender**		
Male	25	(62.5)
Female	15	(37.5)
**Profession**		
Physician	13	(32.5)
Medical assistant	11	(20.0)
Nurse	8	(27.5)
Administrator	8	(20.0)
**Education**		
Nursing preparatory school ^a^	10	(25.0)
Technical institute (Diploma) ^b^	16	(40.0)
College graduate	12	(30.0)
Postgraduate	2	(5.0)

^a^ 3-year study and training after 9th year basic education.

^b^ 2-year study after 12th year basic education.

**Figure 2:**
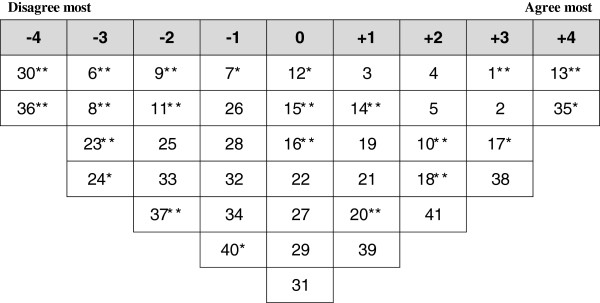
Ideal Q grid for Factor 1 - Content with the current PHC system. * Distinguishing statement significant at <0.05. ** Distinguishing statement significant at <0.01.

**Figure 3:**
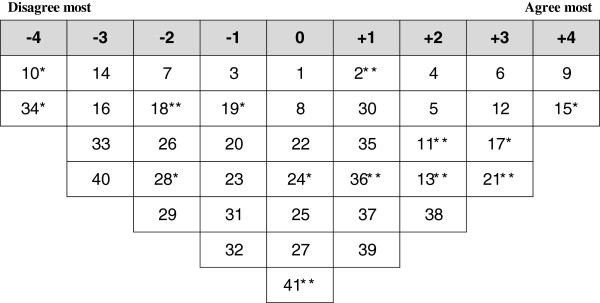
Ideal Q grid for Factor 2 – Professionally-centered viewpoint. * Distinguishing statement significant at <0.05. ** Distinguishing statement significant at <0.01.

**Figure 4:**
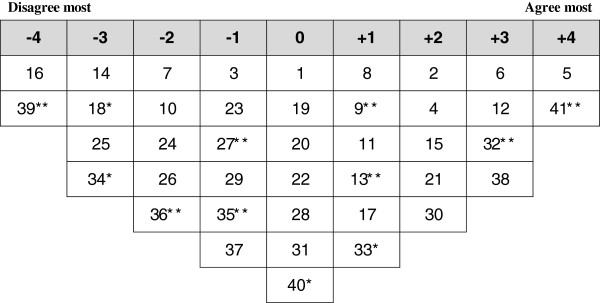
**Ideal Q grid for Factor 3 – Comprehensive perception and problem-based solution.** * Distinguishing statement significant at <0.05. ** Distinguishing statement significant at <0.01.

**Figure 5:**
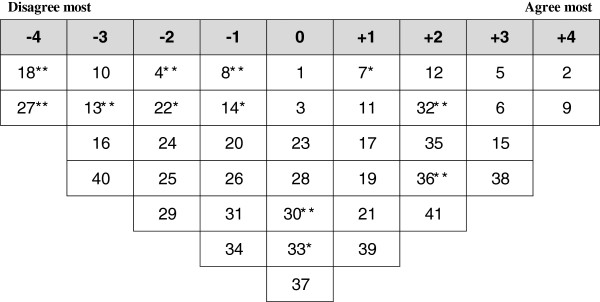
**Ideal Q grid for Factor 4 – Critical to leadership/governance aspects of the system.** * Distinguishing statement significant at <0.05. ** Distinguishing statement significant at <0.01.

Analysis of the participants’ Q-sorts resulted in a four factor solution, i.e. four distinct PHC providers’ viewpoints on the current PHC system (Table 2). One factor emphasized the positive aspects of the current PHC system and three factors highlighted the negative aspects. Ideal Q grids have been generated for each of these factors to clearly illustrate the pattern of response characteristics of each factor (Figures 2, 3, 4 and 5). The four factors were defined by 34 participants (85.0%), whereas two participants did not have a statistically significant load on any of the factors and four participants were confounded, i.e. loaded significantly on more than one factor. The factor loading for each participant on each of the four factors is shown in Additional file 1.

**Table 2:** Statements and factor scores

#	**Statement**	**Factor**			
		**1**	**2**	**3**	**4**
1	PHC centers provide convenient services to poor people	3**	0	0	0
2	PHC centers provide mainly symptomatic treatment to patients	3	1**	2	4
3	Physicians have many privileges and get scientific benefit from working at PHC centers	1	-1	-1	0
4	Referral of patients from PHC centers to specialists is within normal range	2	2	2	-2**
5	Crowding at PHC centers make physicians not having enough time to provide good care for patients	2	2	4	3
6	Weak infrastructure is important barrier to improve the PHC services	-3**	3	3	3
7	Application of family medicine will not control the irrational and repeated visits of patients	-1*	-2	-2	1*
8	Physicians in PHC centers are tired and do not have motivation and energy to provide good care	-3**	0	1	-1**
9	There is lack of appreciation and incentives for PHC providers	-2**	4	1**	4
10	Health education services are provided adequately in PHC centers	2**	-4*	-2	-3
11	PHC centers provide mainly curative services with very little emphasis on prevention	-2**	2**	1	1
12	There is uneven distribution of health staff between PHC centers	0*	3	3	2
13	Statistical reporting and notification of diseases work well in the PHC centers	4**	2**	1**	-3**
14	Necessary investigations are available in PHC centers	1**	-3	-3	-1*
15	Generally, there is irrational use of drugs in PHC centers	0**	4*	2	3
16	PHC providers have sufficient opportunities for training and development courses	0**	-3	-4	-3
17	Provision of partial treatment instead of a full course treatment is an important reason for repeated visits	3*	3*	1	1
18	There is usually appropriate support and planning for PHC centers from DoH	2**	-2**	-3*	-4**
19	Patients maintain a good relation and cooperation with PHC providers	1	-1*	0	1
20	Patients are given enough rights and privacy in the PHC centers	1**	-1	0	-1
21	There is a rapid turnover of physicians in PHC centers	1	3**	2	1
22	Most of laboratory results in PHC centers are accurate	0	0	0	-2*
23	Poor communication between PHC providers and patients is one of the main problems in PHC centers	-3**	-1	-1	0
24	There is a rapid turnover of trained health staff in PHC centers	-3*	0*	-2	-2
25	Introducing higher initial user fees might make some patients, particularly the poor and uneducated, hesitate to visit PHC centers even if they are really ill	-2	0	-3	-2
26	Physicians provide enough care and time to patients	-1	-2	-2	-1
27	There is good follow-up and monitoring of PHC centers from DoH	0	0	-1**	-4**
28	Many PHC providers work according to their private clinic interests	-1	-2*	0	0
29	The DoH has a positive role in facilitating the supply and purchase of materials if regular supplies are not sufficient	0	-2	-1	-2
30	The nursing profession is highly neglected in the PHC centers	-4**	1	2	0**
31	The people in managerial positions in PHC centers are qualified and experienced	0	-1	0	-1
32	There is a need for separating the public and private health sectors	-1	-1	3**	2**
33	PHC centers need to open for longer hours during the day to provide better services	-2	-3	1*	0*
34	There is sufficient use of information technology in PHC centers	-1	-4*	-3*	-1
35	People have easy access to health services at PHC centers	4*	1	-1**	2
36	PHC centers do not have important role in reducing load on hospitals	-4**	1**	-2**	2**
37	The staff in PHC centers are frustrated and poorly motivated due to their low salaries	-2*	1	-1	0
**38**	**Shortage in medication and medical supplies is an important obstacle in providing effective services at PHC centers**	**3**	**2**	**3**	**3**
39	Most patients who visit health centers are really ill	1	1	-4**	1
40	Health services at PHC centers should be provided mainly by general practitioners and there is no need for specialists in health centers	-1*	-3	0*	-3
41	The very low user fees encourage irrational and repeated visits of people to PHC centers	2	0**	4**	2

* Distinguishing statement significant at <0.05.

** Distinguishing statement significant at <0.01.

Bold text indicates consensus statement.

### Factor 1: content with the current PHC system

Seven participants loaded significantly onto factor 1, including three physicians, three administrators and one medical assistant. Five of them were from PHC centers located in Erbil city center. Factor 1 reflected the positive perception toward the efficiency of the current PHC system. Figure 2 illustrates the ideal grid for this factor.

The characterizing and distinguishing statements that were associated with this factor were related to having adequate support and planning for PHC centers from the Directorate of Health (DoH), adequate infrastructure and adequate appreciation and incentives for PHC providers. They were also related to many positive aspects of PHC workforce development like availability of sufficient opportunities for professional development, appreciation of nursing profession, physicians being motivated to provide good care and lack of rapid turnover and uneven distribution of health staff.

This factor was also distinguished by statements that PHC centers provide easy access to health services, have important role in reducing load on hospitals and have properly working statistical reporting and notification of diseases. Other distinguishing aspects of this factor were concerned with having PHC centers providing convenient services to poor people and emphasizing adequately on the preventive health services particularly health education services. This factor was associated with a general satisfaction with the quality of communication between PHC providers and patients and was less concerned about irrational use of drugs. However, it underlined a number of negative aspects of the system including provision of partial treatment and focusing largely on symptomatic treatment.

This factor was unique by having two neutral distinguishing statements about inability of family medicine, if applied, to control irrational visits and that the health services at PHC centers should be provided mainly by general practitioners rather than specialists.

### Factor 2: professionally-centered viewpoint

Eleven participants loaded significantly onto factor 2 including five nurses, four medical assistants, one physician and one administrator. Four of them were from the PHC centers located in Erbil city center, three from the PHC centers close to city and four from the PHC centers remote from the city. Factor 2 emphasized the negative aspects of the PHC system and was concerned mainly with professional issues. Figure 3 illustrates the ideal grid for this factor.

The main concerns associated with this factor were related to the PHC workforce development component of PHC structure. The characterizing and distinguishing statements were related to the lack of sufficient opportunities for professional development, lack of appreciation and incentives for PHC providers and lack of appropriate support and planning for PHC centers from DoH. Other aspects related to this factor included rapid turnover of physicians and health staff at PHC centers and uneven distribution of health staff between PHC centers. Respondents loading on this factor disagreed with the statements that PHC providers work according to their private clinic interests and PHC centers need to open for longer hours during the day to provide better services. They preferred having health services at PHC centers provided by specialists rather than general practitioners.

Another aspect of this factor was related to the poor quality of PHC services including provision of partial treatment, irrational use of drugs and lack of necessary laboratory investigations. According to this factor, the PHC centers provide mainly curative services with little emphasis on prevention particularly health education services. Other problems emphasized by this factor included the weak infrastructure of PHC system and insufficient use of information technology in PHC centers.

This factor had two neutral distinguishing statements. These included having properly working statistical reporting and notification of diseases in the PHC centers and that PHC centers do not have important role in reducing load on hospitals.

### Factor 3: comprehensive perception and problem based solution

Nine participants loaded significantly onto factor 3 including five physicians, two administrators, one medical assistant and one nurse. Four of them were from the PHC centers located in Erbil city and four from the PHC centers remote from the city. Factor 3 comprehensively approached the PHC system, constructively criticized it and provided problems-based solutions to improve it. Figure 4 illustrates the ideal grid for this factor.

The characterizing and distinguishing statements of this factor emphasized irrational use of services as they considered the low user fees an encouraging factor for irrational visits. The resultant crowding makes physicians unable to provide efficient services which may justify the introduction of higher initial user fees to control irrational use of services.

This factor uniquely emphasized provision of health services for longer hours during the day, endorsed separating the public and private health sectors and preferred having PHC centers run by general practitioners rather than specialists.

This factor also emphasized the poor access to health services at PHC centers, poor quality of services particularly that of shortage of medications and medical supplies and unavailability of the necessary investigations. The characterizing and distinguishing statements recognized lack of support and planning from DoH and weak infrastructure. They also highlighted the uneven distribution of health staff and considered PHC providers lacking enough opportunities for professional development. This factor valued the importance of PHC centers on reducing load on hospitals and underlined insufficient use of information technology in PHC centers.

Three distinguishing statements were more neutral compared to other factors. These included lack of appreciation and incentives for PHC providers, having properly working statistical reporting and notification of diseases and having good follow up and monitoring from DoH.

### Factor 4: critical to leadership/governance of the system

Seven participants loaded significantly onto factor 4 including three medical assistants, two physicians, one nurse and one administrator. Four of them were from the PHC centers located in Erbil city. This factor emphasized the negative aspects in the PHC system particularly toward the leadership/governance related issues. Figure 5 illustrates the ideal grid for this factor.

The main concerns of the statements associated with this factor were largely related to poor governance and leadership aspects of the PHC structure. These statements pointed out with great certainty the inappropriate support and planning from DoH, poor follow up and monitoring of PHC centers from DoH, poor infrastructure, lack of appreciation and incentives to PHC providers, lack of sufficient opportunities for professional development and poor statistical reporting and notification of diseases. They were also concerned with the inefficient PHC referral system. The respondents loading on this factor preferred having PHC centers run by specialists rather than general practitioners. This group also emphasized other problems related to poor service delivery including inadequate provision of health education services, irrational use of drugs and focusing mainly on symptomatic treatment.

These respondents were unique in their neutrality about the idea that nursing profession being highly neglected in PHC centers, availability of necessary investigations in PHC centers and the need for PHC center to open for longer hours to provide better services.

### Consensus statement

There was only one statement of consensus with no significant difference in scores across the four factors, which was related to shortage of medications and supplies in PHC centers (Table 2).

## Discussion

A positive viewpoint and three different negative viewpoints among PHC providers towards the Iraqi PHC system were identified in this explorative study. Reporting the presence of differences in viewpoints concerning the Iraqi PHC system among PHC providers might not be totally new knowledge by itself. However, this study was able to identify and characterize these differences in a novel and insightful way. It is certainly difficult to argue at this stage that this is the definitive range and variety of viewpoints among the PHC providers on the basis of one explorative study. However, we consider these viewpoints to be representative of the range and varieties of viewpoints existing among PHC providers in Erbil governorate and Iraqi Kurdistan region in general.

As PHC providers have a pivotal role in delivering PHC services, they can be considered “street-level bureaucrats” who are confronted with real world challenges in the primary care sector, yet face inadequacies of under-funded government systems. Superficially, street-level bureaucrats constitute a level of implementation of public policies as they are tasked with ensuring policies are carried out. Yet as individuals, PHC providers represent a small-scale level of policy making. They decide the specific operation and execution of policies. On a larger scale, the combined actions and decisions of street-level bureaucrats in their bureaucracy amalgamate to form an agenda and heavily influence the direction of policy [29]. Therefore, the viewpoints of study participants can provide valuable information to health policy makers and PHC managers to direct actions to improve the PHC system and the related health services.

This study identified a number of positive aspects of the current PHC system of Iraq but highlighted enormous problems the system faces as illustrated by the characterizing and distinguishing statements of the different identified factors. Many of these aspects correspond well with the findings of the focus groups [9] and open-ended questionnaire survey [24] that preceded this study as well as with the limited relevant research from Iraq. The relatively easy accessibility and provision of convenient services to the poor were also reported by three other studies from Iraq [24,25,30]. Easy access to PHC facilities is also a common feature in settings similar to Iraq. For example, a study from Egypt revealed easy accessibility of PHC facilities with majority of patients (58%) reaching the facility in less than 10 minutes [31]. Another study from Iran showed a high clients’ satisfaction with access to PHC with a high proportion of patients (51.3%) accessed the PHC centers by walking [32].

The problems related to poor service delivery, particularly irrational use of services, irrational treatment, poor provision of health education and poor referral system, were also reported by the focus groups study of the PHC providers that preceded this study [9]. While the coverage of PHC in Iran has substantially increased, improving the quality of care remained one of the main concerns in PHC especially in urban areas [33]. In Iran again clients’ satisfaction with continuity of care, comprehensiveness of care and provision of health educational materials was much lower than other aspects of access to services and effectiveness of care [32]. A study from Jordan revealed an increase in client visits to PHC centers resulting in longer waiting times and sometimes necessitating a return visit and shorter provider-patient contact affecting both the quality of service and client satisfaction [34]. The low user fees as a main reason for repeated and irrational visits to PHC centers and increasing such fees to prevent irrational visits remain a matter of debate. Experience from a number of African countries showed that introducing or increasing user fees impose a heavier burden on the poor who are most likely facing a high burden of disease [35].

Problems related to workforce development including uneven distribution, rapid turnover and lack of professional development opportunities were also reported by two other studies from Iraq [13,36]. In Turkey, primary care physicians are unevenly distributed provincially [37]. Few opportunities for professional development of primary care providers were also reported in Serbia [2].

The general preference for the establishment of a family medicine system has been emphasized by several studies from Iraq [9,11,13,25]. Introduction of the specialty of family medicine as the population’s first line of care and adapting the current PHC centers into family health centers staffed with physicians trained in family medicine was similarly recognized in Serbia as a main priority to improve the organization of the primary care in Serbia [2].

To the best of our knowledge this is the first Q-study in Iraq. However, we found it a feasible research method and useful way to analyze PHC providers’ viewpoints towards the PHC system. The PHC providers found it interesting to participate in the study and were confident in ranking the statements. During the ranking of the statements the participants remained involved and willing to discuss different statements and issues related to the system. Based on this unique experience of using Q-methodology in the Iraqi context, we think that this methodology is a useful and practical tool for future health system research and studying the perspectives of different actors in the health system. However, this study involves a number of limitations. Due to the small number of participants included in studies employing Q-methodology, generalization is limited and finding out the proportion of respondents that hold particular viewpoints is not possible [38,39]. However, generalization in this study was not intended as Q-methodology is exploratory in nature that can provide a useful insight to the available viewpoints in the society and characterization of each viewpoint. It might also provide an initial understanding of the sociodemographic characteristics associated with each viewpoint. As a hypothesis generating tool, Q-methodology can be followed up with larger surveys to examine these uncovered viewpoints and associated factors [40].

The results of Q-studies depend to a certain degree on the methods and the model used to develop and structure the representative statements and interpret the factors. In this study, focus groups of PHC providers [9] and open questionnaire survey of PHC managers, policy makers and academics [24] were used to develop the study concourse while a modified version of the WHO conceptual framework of health system building blocks was used to develop and structure the representative statements [26]. The WHO conceptual framework of health system building blocks was adopted because it is a valid and widely used framework for health system studies in addition to be generally applicable and sufficiently comprehensive to suit well the purpose of this study. The initial aim was to use this framework to interpret the factors, however due to the diversity of viewpoints, this framework was of benefit in interpreting factor 4 only. Thus, lack of a reliable framework for interpreting all the factors is considered a limitation of this study. A number of alternative methods for concourse development and different models for developing and structuring statements and interpreting factors related to health system could have been used that might have resulted in different findings. However, this should not be considered an issue of great concern as the limited number of comparative studies that have been carried out indicate that different sets of statements structured in different ways can nevertheless be expected to come with the same conclusions [41]. Being new to this field we did not use the opportunity of conducting follow-up interviews to obtain more information. Such interviews could have been helpful in interpreting and describing the statistical results and might have provided us with more insights into the relation between viewpoints and the experience of PHC providers from different backgrounds.

## Conclusion

This study revealed a range of diverse viewpoints of PHC providers toward the Iraqi PHC system and recognized the issues associated with each viewpoint. These findings can contribute to a better understanding of health policy makers and PHC managers of PHC providers’ concerns about the PHC system and might contribute to change in management. This study helped identifying the main issues of concern to this important group of stakeholders in terms of problems the system faces and the priority needs for its improvement. Information on the PHC providers’ viewpoints on the system can lead to better performance of the system through further exploring the issues raised by the respondents.

## Abbreviations

PHC: Primary health care; DoH: Directorate of health.

## Competing interests

The authors declare that they have no competing interests.

## Authors’ contributions

NPS, NGAT and TSAH conceptualized the study. NPS, NGAT and ES designed the study. NPS and NGAT collected the data. NPS and ES carried out data analysis and interpretation. NPS, NGAT and TSAH prepared the manuscript. ES and TSAH extensively reviewed and edited the manuscript. All authors read and approved the final manuscript.

## Pre-publication history

The pre-publication history for this paper can be accessed here:

http://www.biomedcentral.com/1472-698X/13/18/prepub

## Supplementary Material

Additional file 1 **Participants’ characteristics and factor loading on the five factors. **Bold type indicates significant loadings. Significance at the 1% level is taken as a factor loading greater than (2.58 × 1√n), where n = the number of statements - so in this case significant loadings are those higher than 0.403. ^X^ indicates defining sorts. Click here for file
