# Supplementary material for: The range and diversity of providers’ viewpoints towards the Iraqi primary health care system: an exploration using Q-methodology
Source: BMC Int Health Hum Rights. 2013 Mar 21;13:18. doi: 10.1186/1472-698X-13-18 (PMC3606603; doi:10.1186/1472-698X-13-18)
Supplement: Additional file 1 — Participants’ characteristics and factor loading on the five factors. Bold type indicates significant loadings. Significance at the 1% level is taken as a factor loading greater than (2.58 × 1√n), where n = the number of statements - so in this case significant loadings are those higher than 0.403. X indicates defining sorts. [file 1472-698X-13-18-S1.doc]

# Additional files

Additional file 1 – Participants’ characteristics and factor loading on the five factors

| **Participant**  **No.** | **Type of PHCC** | **Gender** | **Age (years)** | **Profession** | **Experience (years)** | **Factor** | | | |
| --- | --- | --- | --- | --- | --- | --- | --- | --- | --- |
|  |  |  |  |  |  | **1** | **2** | **3** | **4** |
| P1 | Close to city | Male | 33 | Physician | 2 | -0.072 | 0.370 | **0.571^x^** | 0.241 |
| P2 | Close to city | Male | 35 | Physician | 2 | -0.209 | 0.224 | **0.499^x^** | 0.279 |
| P3 | Close to city | Male | 46 | Medical assistant | 25 | -0.010 | **0.666^x^** | -0.081 | 0.069 |
| P4 | City center | Female | 29 | Nurse | 1 | 0.208 | **0.471^x^** | 0.187 | 0.277 |
| P5 | City center | Male | 36 | Medical assistant | 6 | **0.417** | **0.476** | 0.135 | 0.117 |
| P6 | City center | Male | 38 | Nurse | 5 | -0.077 | 0.397 | **0.432 ^x^** | 0.337 |
| P7 | City center | Female | 34 | Physician | 2 | **0.434 ^x^** | -0.396 | -0.119 | 0.195 |
| P8 | City center | Male | 58 | Medical assistant | 9 | **0.560^x^** | 0.268 | 0.032 | 0.299 |
| P9 | City center | Male | 47 | Administrator | 25 | **0.580^x^** | 0.041 | 0.252 | -0.167 |
| P10 | City center | Male | 40 | Medical assistant | 8 | 0.078 | 0.251 | 0.385 | **0.527^x^** |
| P11 | City center | Female | 32 | Physician | 2 | **0.476^x^** | 0.100 | 0.199 | -0.212 |
| P12 | City center | Female | 41 | Physician | 9 | -0.60 | 0.081 | -0.065 | **0.463^x^** |
| P13 | City center | Male | 35 | Physician | 5 | -0.149 | -0.104 | 0.211 | **0.541^x^** |
| P14 | City center | Male | 45 | Medical assistant | 11 | 0.192 | 0.401 | 0.369 | **0.412^x^** |
| P15 | City center | Female | 22 | Administrator | 1 | **0.601^x^** | -0.196 | -0.157 | -0.040 |
| P16 | City center | Female | 30 | Physician | 1 | 0.250 | 0.023 | 0.**528^x^** | 0.150 |
| P17 | City center | Male | 45 | Administrator | 20 | 0.044 | **0.507^x^** | 0.353 | 0.292 |
| P18 | City center | Male | 32 | Physician | 1 | -0.143 | **0.411^x^** | 0.248 | 0.133 |
| P19 | Close to city | Female | 28 | Physician | 3 | **0.582** | -0.033 | 0.3999 | 0.025 |
| P20 | Close to city | Male | 29 | Physician | 3 | **0.414** | 0.029 | **0.744** | -0.026 |
| P21 | Close to city | Male | 25 | Medical assistant | 1 | 0.104 | 0.186 | **0.736^x^** | 0.258 |
| P22 | Close to city | Male | 56 | Administrator | 20 | **0.627^x^** | 0.059 | -0.036 | -0.213 |
| P23 | Close to city | Male | 30 | Administrator | 5 | 0.398 | 0.178 | -0.268 | 0.160 |
| P24 | Close to city | Male | 43 | Medical assistant | 20 | 0.077 | **0.442^x^** | 0.353 | 0.359 |
| P25 | Close to city | Female | 32 | Nurse | 9 | -0.182 | **0.588^x^** | 0.182 | -0.049 |
| **Participant**  **No.** | **Type of PHCC** | **Gender** | **Age (years)** | **Profession** | **Experience (years)** | **Factor** | | | |
|  |  |  |  |  |  | **1** | **2** | **3** | **4** |
| P26 | Close to city | Male | 40 | Medical assistant | 18 | 0.207 | 0.193 | 0.262 | 0.305 |
| P27 | Close to city | Male | 42 | Medical assistant | 13 | 0.088 | 0.206 | 0.350 | **0.440^x^** |
| P28 | Close to city | Female | 25 | Administrator | 1 | -0.228 | 0.217 | 0.059 | **0.465^x^** |
| P29 | Close to city | Male | 26 | Physician | 1 | -0.080 | 0.071 | **0.595** | **0.420** |
| P30 | Close to city | Female | 34 | Medical assistant | 7 | 0.248 | **0.638^x^** | 0.064 | 0.279 |
| P31 | Remote from city | Male | 47 | Administrator | 13 | 0.300 | 0.205 | **0.647^x^** | 0.076 |
| P32 | Remote from city | Female | 30 | Nurse | 22 | 0.360 | **0.462^x^** | 0.042 | 0.213 |
| P33 | Remote from city | Female | 24 | Medical assistant | 1 | 0.027 | **0.460^x^** | 0.291 | -0.158 |
| P34 | Remote from city | Male | 28 | Physician | 3 | 0.083 | 0.079 | **0.568^x^** | 0.048 |
| P35 | Remote from city | Male | 28 | Physician | 1 | -0.212 | 0.347 | **0.772^x^** | -0.019 |
| P36 | Remote from city | Male | 38 | Nurse | 17 | 0.020 | **0.494** | **0.544** | 0.047 |
| P37 | Remote from city | Male | 48 | Administrator | 25 | -0.194 | 0.221 | **0.595^x^** | 0.091 |
| P38 | Remote from city | Female | 35 | Nurse | 10 | 0.238 | 0.353 | 0.328 | **0.440^x^** |
| P39 | Remote from city | Female | 25 | Nurse | 7 | 0.168 | **0.455^x^** | 0.321 | 0.313 |
| P40 | Remote from city | Female | 24 | Nurse | 6 | 0.073 | **0.672^x^** | 0.297 | 0.375 |
| Eigenvalues | | | | | | 3.3 | 10.5 | 2.4 | 1.6 |
| Defining sorts | | | | | | 7 | 11 | 9 | 7 |
| Explained variance % | | | | | | 8 | 26 | 6 | 4 |
| Explained variance cumulative % | | | | | | 8 | 34 | 40 | 44 |

Bold type indicates significant loadings. Significance at the 1% level is taken as a factor loading greater than (2.58 x 1√n), where n= the number of statements - so in this case significant loadings are those higher than 0.403.

^X^ indicates defining sorts.
